# Information integration and decision making in flowering time control

**DOI:** 10.1371/journal.pone.0239417

**Published:** 2020-09-23

**Authors:** Linlin Zhao, Sarah Richards, Franziska Turck, Markus Kollmann

**Affiliations:** 1 Institute of Mathematical Modeling for Biological Systems, Heinrich-Heine-University Dusseldorf, Dusseldorf, Germany; 2 Institute of Population Genetics, Heinrich-Heine-University Dusseldorf, Dusseldorf, Germany; 3 Max-Planck Institute for Plant Breeding Research, Cologne, Germany; Universidad Miguel Hernández de Elche, SPAIN

## Abstract

In order to successfully reproduce, plants must sense changes in their environment and flower at the correct time. Many plants utilize day length and vernalization, a mechanism for verifying that winter has occurred, to determine when to flower. Our study used available temperature and day length data from different climates to provide a general understanding how this information processing of environmental signals could have evolved in plants. For climates where temperature fluctuation correlations decayed exponentially, a simple stochastic model characterizing vernalization was able to reconstruct the switch-like behavior of the core flowering regulatory genes. For these and other climates, artificial neural networks were used to predict flowering gene expression patterns. For temperate plants, long-term cold temperature and short-term day length measurements were sufficient to produce robust flowering time decisions from the neural networks. Additionally, evolutionary simulations on neural networks confirmed that the combined signal of temperature and day length achieved the highest fitness relative to neural networks with access to only one of those inputs. We suggest that winter temperature memory is a well-adapted strategy for plants’ detection of seasonal changes, and absolute day length is useful for the subsequent triggering of flowering.

## Introduction

**Fig 1:**
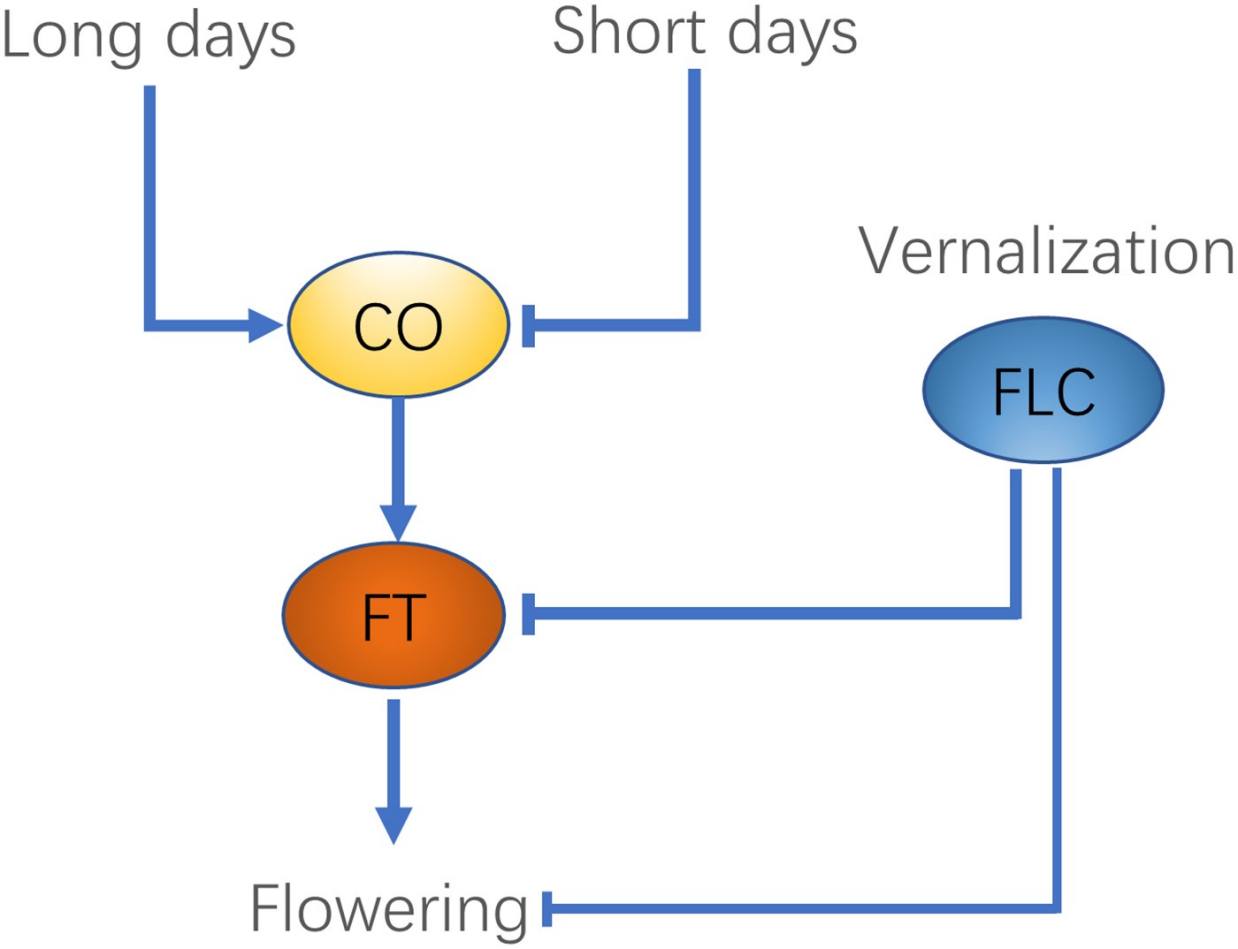
Flowering time regulation in *Arabidopsis thaliana*. In *Arabidopsis Thaliana*, long days promote the expression of FLOWERING LOCUS T (FT). The vernalization process also promotes its expression by turning-off its repressor FLOWERING LOCUS C (FLC).

Plants must make correct flowering time decisions in a noisy environment in order to successfully reproduce. As key environmental signals, day length and temperature are processed by plants’ genetic networks for detecting seasonal changes. The core genes and their interplays have been well understood in the model plant, *Arabidopsis thaliana* [1, 2], as shown in Fig 1. The gene *FLOWERING LOCUS T (FT)* merges signals from both day length and temperature, and its encoded protein eventually induces the flowering [3]. The expression of FT genes is promoted by the expression of the gene *CONSTANS (CO)*, whose gene products are produced about 12 hours after dawn and quickly degrade in the dark [1, 3]. Thus, the condition that day length is long enough to produce stable CO proteins is necessary for initiating the flowering of the so-called long-day plants [1, 3, 4]. In particular, for winter annuals of *Arabidopsis thaliana* [5, 6], vernalization is required to cease expression of *FlOWERING LOCUS C (FLC)*, the inhibitor of *FT*. Vernalization involves the exposure of plants to a prolonged period of cold temperature, which induces histone modifications on the epigenetic level for silencing *FLC* [7–9]. The repressed *FLC* allows *FT* to be expressed under long days. Similarly, in perennial *Arabis alpina*, the orthologs of *FLC*, *PERPETUAL FLOWERING 1 (PEP1)*, downregulates the orthologs of *FT*, *AaFT1* and *AaFT3*, and needs to be silenced by vernalization in order for the perennials to flower in the right time [6]. Moreover, for perennial *Arabidopsis halleri*, Nagano et.al [10] demonstrated the role of cooperation between the oscillations of temperature and day length in adaptation to seasonal changes. Thus, the integration of signals from temperature and day length is crucial for both annual and perennial plants to make flowering decisions. Despite the qualitative understanding of the genetic regulation of flowering time, it is unclear from an information point of view why plants have evolved vernalization from fluctuating winter temperatures and how it relates to day length in flowering decisions.

Many theoretical studies have contributed to the understanding of the vernalization mechanism. It was shown to be an inheritable and stable epigenetic switch for the expression of *FLC* [11–15]. Dodd et.al [15] developed a stochastic model for *Schizosaccharomyces pombe* showing that gene expressions bistability can be established by accumulating histone modifications, which acted as an epigenetic memory. The work of Angel et al. [9] extended their approach by incorporating histone modifications of *FLC* in *Arabidopsis thaliana* and investigating how the different epigenetic states could be controlled. Several studies have reported that FLC repression was cell-autonomous and that cold temperature memory was encoded by the fraction of cells with repressed FLC [8, 9, 16, 17]. Due to the positive feedbacks that lead to adding more of the same type of histone modifications, a particular cell would mostly have one type of modifications at *FLC*. To understand how plants utilize fluctuating temperature in vernalization, Antoniou-Kourounioti et al. developed a model by incorporating thermosensing on multiple timescales and suggested that the sensing was broadly distributed in plants [18].

Investigations of flowering time regulation in *Arabidopsis* exploited mathematical modeling and experiments. Wilczeck et al. developed a model to incorporate the impacts of temperature, day length, and vernalization on flowering initialization in different accessions of *Arabidopsis thaliana* [5, 19]. Their analyses yielded a photothermal measure of plant states which was able to accurately predict flowering time of *Arabidopsis Thaliana* field plants. Another dynamic model described interplays between *FT* and *FLC* in *Arabidopsis halleri* to study impacts of temperature on flowering decisions [20]. It was able to reproduce the observed seasonal expression changes and estimate the climate change-induced reduction of flowering season. Hepworth et al. reported that the spikes of temperatures above 15°C may have deleterious consequences for vernalization [21]. These studies have significantly refined our understanding of the effects of temperature and day length on *Arabidopsis* flowering, but the mechanisms employed by a variety of plants in various environments cannot be described by the same processes [23]. A method leading to a more general understanding of climate information processing would promote understanding of flowering-time decision making for a greater variety of plants.

Our study focuses on the extraction of available information from climate data (temperature and day length) and its usefulness in making precise flowering decisions. We first established a simple stochastic model for vernalization in perennials, which showed that the idealized expression patterns of *FT* or *FLC* can be reconstructed for temperate climates due to exponentially decaying correlation in temperature fluctuations. The stochastic model does not apply to other climates where temperature fluctuations correlate differently. To relax this restriction on climate properties, we employed artificial neural networks to learn idealized gene expression patterns from several climate datasets. We showed that, in temperate city Cologne, the neural network models trained solely on temperature memory roughly reconstructed the idealized expression patterns of *FLC*. However day length data was required to resolve the danger of incorrect flowering time decisions based on a local optimum in September rather than April. Further, to simulate the evolutionary adaptation to environmental conditions, individual neural networks were used in a simulation of evolution for plants with access to temperature, day length, or both. Simulations with different mutation rates and population sizes showed a persistent selective advantage for the neural networks with access to the combined temperature and day length data.

## Materials and methods

### Datasets

The temperature and day length data of several climate regions (Table 1) were retrieved from NOAA [24] and PTAFF [25]. Temperatures were recorded as the daily maximum and minimum, and data from different stations are considered distinct. The mean of daily maximum and minimum is regarded as the daily average temperature. To account for the effect of noise in daily light quantity [26–28] on the day length, Gaussian noise was used to corrupt the day length data and simulate real variations due to weather conditions.

**Table 1:** Selected regions and cities for collecting climate data.

Cold Regions	Obvious Seasonal Changes	Less Seasonal Changes
Oslo	Cologne	Kahului
	Auckland	San Francisco

### Master equation and Hermite polynomial

Chemical master equations are used to model the probabilistic states of chemical reactions over time [29–31]. Following the previous work of modeling birth-death process [32], for the reaction $\phi \overset{\beta }{\underset{\lambda }{⇌}}\text{B}$, the probability *p* of having *n* molecules of *B* can be described as Eq (1) for time *t*.

$$\begin{array}{c} \partial_{t}p\left(n,t\right)=\beta (p(n-1,t)-p(n,t))-\lambda np\left(n,t\right)+\lambda \left(n+1\right)p\left(n+1,t\right) \end{array}$$ (1)

In equilibrium, *p*(*n*) is Poisson distributed (see Supplement Section 3.1 in S1 File). In our study, a modified form of this model was used with B representing the cellular state of having active modifications at the *FLC* locus. Different from the basic model, the production rate is adapted to be temperature dependent. Since the temperature recordings are time series, the temperature dependence of the model make it able to integrate temperature properties. This modification necessitates the use of Hermite polynomials [33, 34] to solve the master equation (see Supplement Section 3.1 in S1 File).

The original daily average temperature from different regions have been used for the analytical deductions based on the master equation.

### Neural network models and data features

Artificial neural networks was used as a complementary method to extract information from temperature and day length. A feedforward neural network is comprised of a number of neurons to transmit information in only one direction, from the input data through hidden neurons to output neurons. Each neuron can be regarded as either a computing node or a decision maker which outputs a decision by weighing and transforming the information it receives from upstream neurons. A detailed formal description of neural networks is in Supplementary section 4 in S1 File.

Fully connected feed-forward neural networks with one hidden layer were used to classify different time windows in each year and regress the idealized expression patterns of *FT* and *FLC* in Arabidopsis perennials.

For the classification, each year was shrank to 360 days for simplicity. For example, it will be divided into 12 windows corresponding to 12 months if the window size is set to 30 days. The neural networks then need to determine which month the input window belongs to, given 30 days of temperatures within the window. The daily temperatures were summarized by daily maximum and minimum.

**Fig 2:**
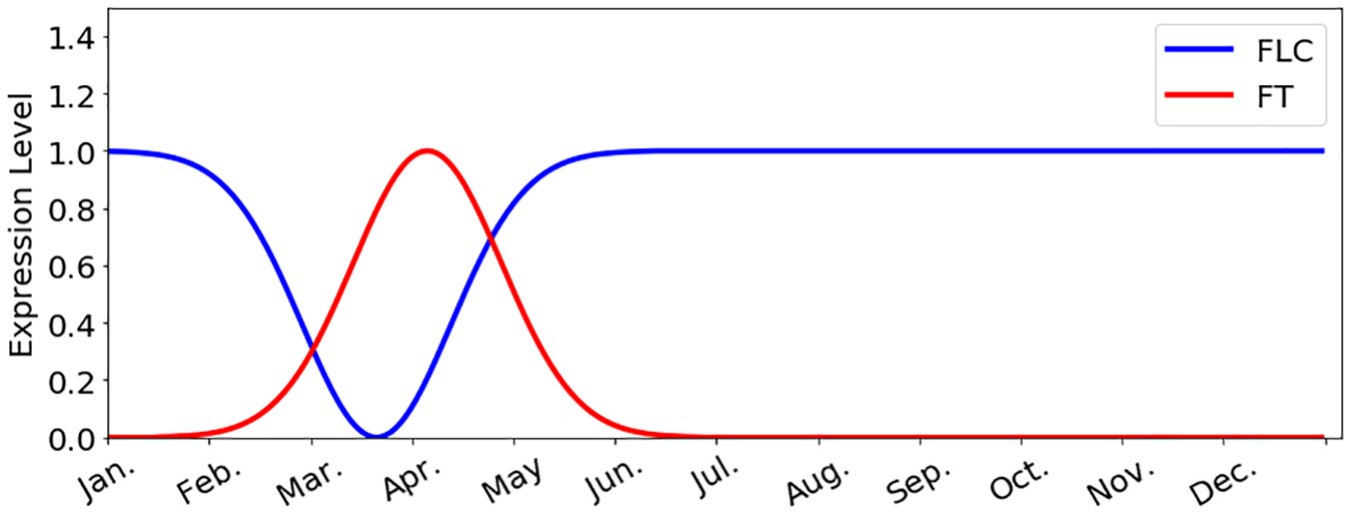
Idealized gene expressions. The idealized expression levels of *FLC* and *FT* for *Arabidopsis perennials* in the northern hemisphere.

For the regression of gene expressions, the target can be idealized patterns of *FT* or *FLC*. Specifically, the expression pattern of *FT* is characterized by a normal distribution *p*(*t*), peaking in April every year as shown in Fig 2, where *t* denotes certain day of a year. And the *FLC* is featured as an upside-down normal distribution centered at middle of March [35], which is 15 days earlier than the peak of *FT* [20]. To learn the expression patterns from the climate data, the input features consist of daily temperature maxima and minima and day lengths of the past days. That is, the expression level on a specific day is determined by the plant’s memory of temperature and day length of the past days.

The Neural Networks Toolbox in Matlab [36] was used to build the classification and regression models.

### Evolution of individual neural networks

Plant selection pressure and evolution were simulated using neural networks to represent individual plants, with weights of individual networks analogous to plant genotypes (see details in Supplement Section 5 in S1 File). A group (population) of networks trained on distinct subsets of the same climate data were used to evaluate the flowering decision-making strategies of plants based on climate information. There were three populations in each simulation, each with access to only temperature, only day length, or both. Each generation of individuals was trained on randomly selected subsets of climate data from Cologne. At the end of each reproductive cycle, fitness was measured by the Kullbach-Leibler distance between target and neural network-predicted gene expression level, and mutations (in the form of neural network weight perturbations) were introduced at specific rates which varied across simulations for the next generation. The simulation procedure is summarized in S8 Fig in S1 File. It was implemented in Python 2.7, and the Theano package [37] was used to optimize individual networks. The results of each simulation were reported as the proportion of individuals from each of the three populations throughout the entire simulation, which would be greater for neural networks with higher fitness leading to better reproductive odds.

## Results

### Expression patterns reproduced from modeling key reactions

Plants need to avoid the disastrous effect of flowering at the wrong time as a reaction to sudden and sustained temperature fluctuations. We hypothesize that long-cold based vernalization is not only for capturing the winter cold temperature but also for canceling this effect and sensing the winter robustly. The vernalization machinery can be interpreted that plants have evolved biochemical processes to capture and accumulate the information in cold temperature that is a reliable signal in temperate regions. Inspired by the machinery, we modeled the process of having *n* cells with active histone modifications, which is driven by real temperature to reconstruct the idealized expression patterns of *FLC/FT* of *Arabidopsis* perennials.

**Fig 3:**
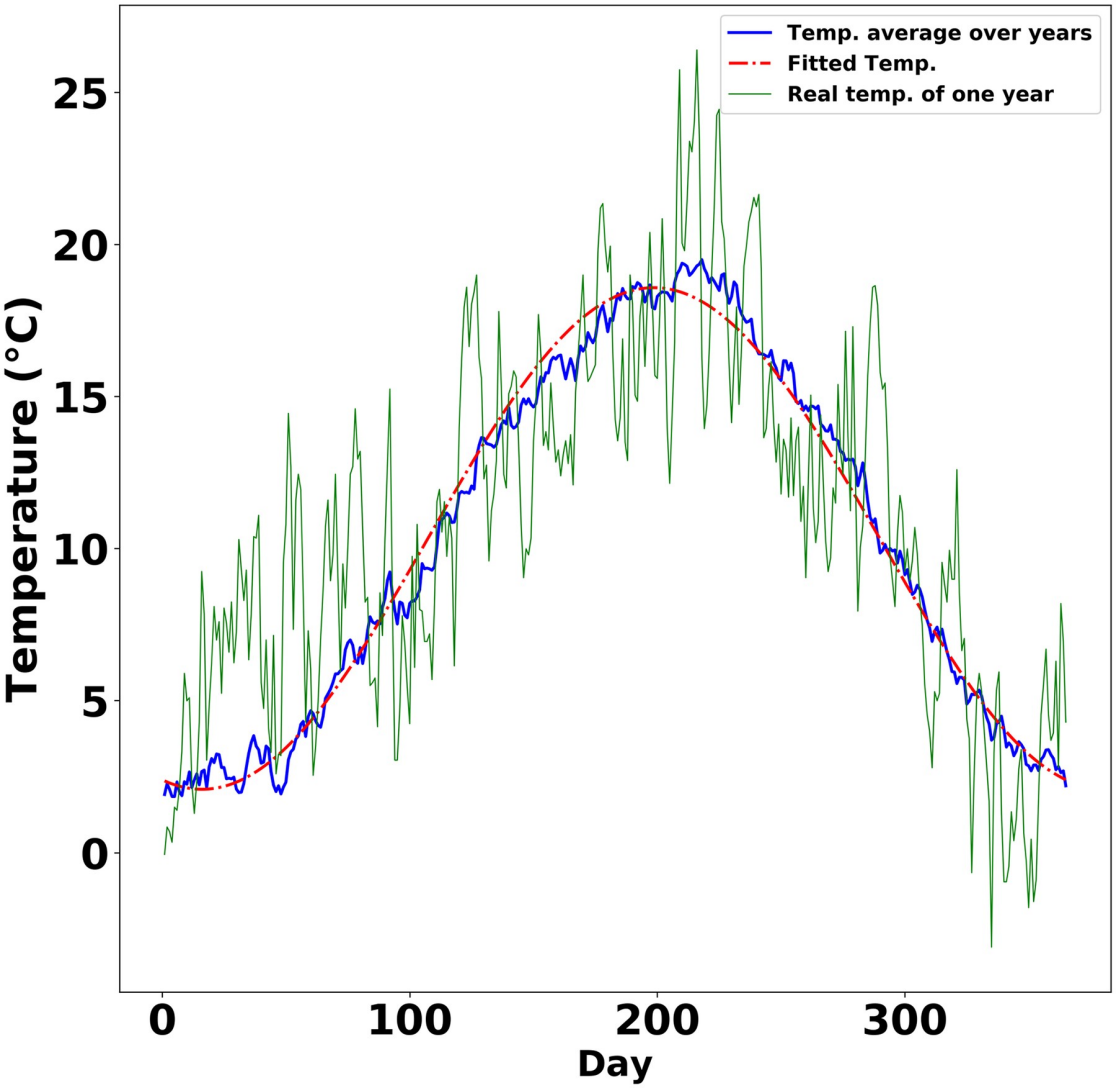
Temperature dynamics in Cologne. The temperature dynamics consist of the seasonal changes and the daily temperature fluctuations, which were fitted by a second order Fourier series. The dynamical data were obtained by averaging 93 years of temperatures in temperate city Cologne. The first day in the plot was January 1st.

**Fig 4:**
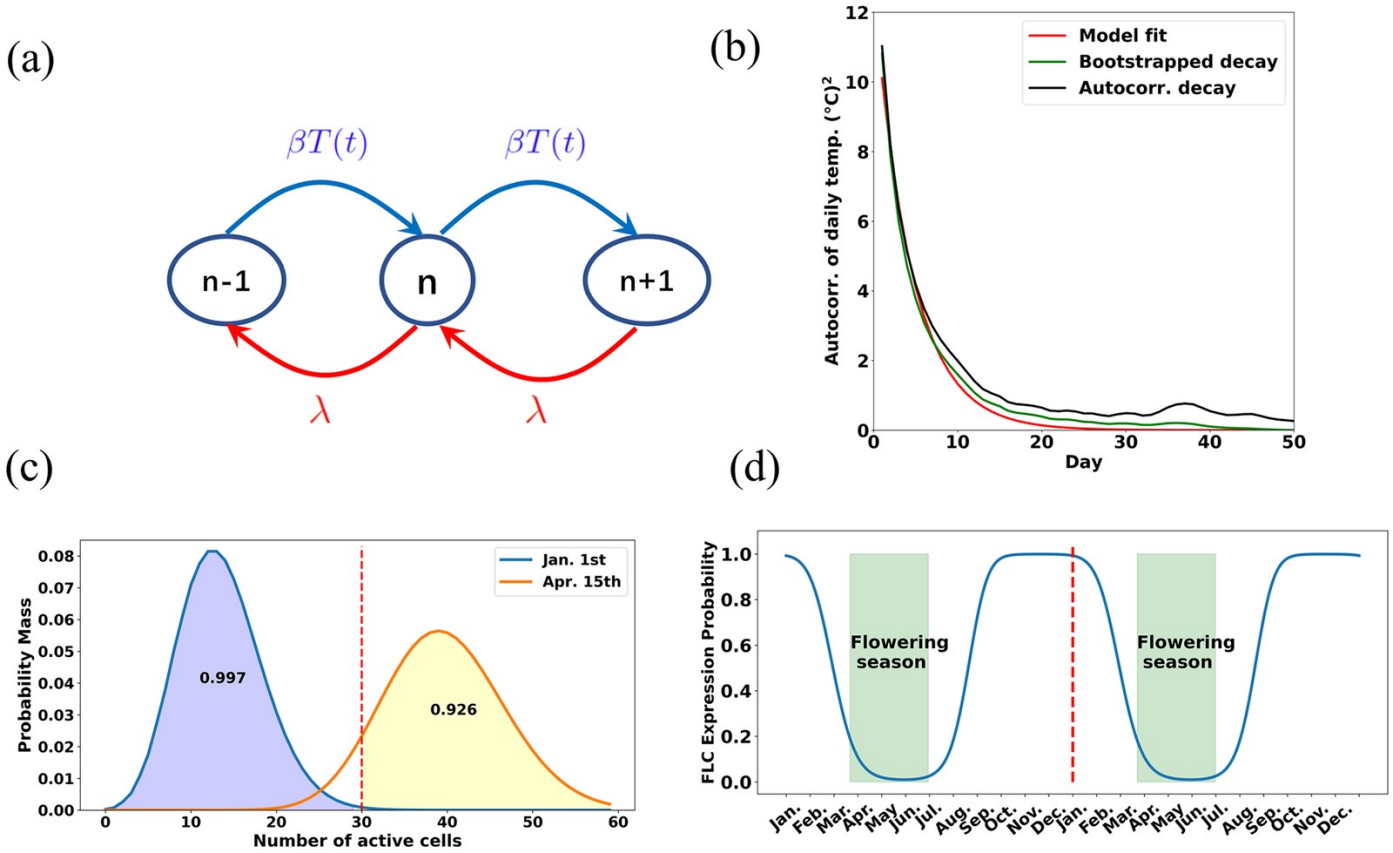
Reconstruct the switch behavior of *FLC*. (a): The vernalization was simplified as birth-death process for actively modified cells. *n* stands for the number of active cells, the production rate *βT*(*t*) depends on the temperature *T*(*t*) and λ is the degradation rate. Solving the model led to the distribution *p*(*n*, *t*) which is parameterized by *β* and λ; (b): For the data from Cologne, the autocorrelation in daily temperature decays exponentially, the bootstrapping was performed by using block length of 50 days; (c): The probability *p*(*n*, *t*) distribution over number of active cells (in total 60 cells in the simulation) on 1st January and 15th April; (d): The switching *FLC* expression behavior was constructed from the probabilities of having less than 30 active cells over two years, with green areas for potential flowering seasons.

In the following, we denoted daily average temperature as *T*(*t*) for day *t* ∈ [1, ⋯, 365]. To investigate the seasonal changes and fluctuations in real temperature, it is decomposed into three parts as $T\left(t\right)=\bar{T}+\langle T\left(t\right)\rangle +\delta T\left(t\right)$ with $\bar{T}$ denoting the average yearly temperature, 〈*T*(*t*)〉 the seasonal temperature changes, and *δT*(*t*) the remaining temperature fluctuations. The temperature dynamics comprising of 〈*T*(*t*)〉 and *δT*(*t*) are shown in Fig 3 for Cologne. The Fourier fitting of 〈*T*(*t*) was detailed Supplementary section 2 in S1 File. The temperature fluctuation on a specific day typically correlates with its neighboring days. And the longer periods of unseasonally cold or warm temperatures may confuse the plants more than the shorter periods. Therefore, we analyzed the autocorrelation times, which quantify the correlation length in a time series, in the fluctuations of temperature from five different regions (Table 1). The autocorrelation times in Cologne, Auckland and Oslo decay exponentially, and decay faster in Auckland than in Cologne and Oslo (Supplementary Section 2 in S1 File, S3a, S3b Fig in S1 File). This is consistent with experimental observations that some plants in Auckland required only two weeks of vernalization [38], while vernalization requires typically around 6 weeks in Cologne [5] and even three months in North Sweden [39]. Having verified the exponential decay of temperature fluctuations, without loss of generality, we used the climate data of Cologne for the successive modeling, where the autocorrelation in averaged yearly-cycle temperature fluctuations decays exponentially with an approximate half life of 4 days (Fig 4b). To further evaluate the exponential fitting of the fluctuation decay, the bootstraping of temperature data with block length of 50 showed that the fitted coefficients of the exponential function indeed located in the bootstrapped confidence interval(further details in Supplementary Section 2 in S1 File).

To investigate the effect of temperature dynamics on vernalization, we modeled the number of cells which have their histones all with active modifications as a death-birth process. The assumption of having all active modifications in a cell relied on the fact that the fraction of histones in one state affects modifications in their own vicinity, which makes histone modification a relative fast process. As shown in Fig 4A, the probability of having *n* active cells at time *t* is denoted as *p*(*n*, *t*):

$$\begin{array}{c} \partial_{t}p\left(n,t\right)=\beta T\left(t\right)(p(n-1,t)-p(n,t))-\lambda np\left(n,t\right)+\lambda \left(n+1\right)p\left(n+1,t\right), \end{array}$$ (2)

with *βT*(*t*) the temperature dependent production rate and λ the temperature-independent degradation rate. Due to the fact that the autocorrelation time of temperature fluctuations decays exponentially, the stochastic differential equation has a concise form of solution given by

$$\begin{array}{c} p\left(n,t\right)=e^{a-b}\frac{\alpha^{n}}{n!}H_{n}(\frac{b+2\alpha^{2}}{2\alpha }). \end{array}$$ (3)

with $a≔\frac{\beta^{2}\sigma^{2}}{2\lambda (\lambda +\tau^{-1})}$ and $b=\frac{\bar{T}\beta }{\lambda }+\beta D\left(\lambda ,t\right)$. Here, we denoted by $D\left(\lambda ,t\right)=\int_{-\infty }^{t}\langle T\left(t\right)\rangle e^{-\lambda (t-t^{\prime })}dt^{\prime }$ the expected memorized temperature. The parameters *σ*^2^ and *τ* denote the averaged variance and autocorrelation time of temperature fluctuations respectively. *H*_*m*_(⋅) denotes the *m*th Hermite polynomial. The parameterization for *a*, *b*, and *α* is detailed in the S1 File.

To reconstruct the expression patterns, we need to define an objective for getting the optimal reaction rates based on the probability *p*(*n*, *t*). We reduced the number of cells for computational feasibility and assumed that *N*_max_ = 60 cells were available and *N*_*c*_ = 30 cells were sufficient to turn off the expression of *FLC* on the plant level. The objective function of flowering probability in time window between March and June and non-flowering probability between July and next February can be defined as

$$\begin{array}{c} F\left(\beta ,\lambda \right)=\int_{Mar}^{Jun}\sum_{n=N_{c}}^{N_{max}}p\left(n,t\right)dt+\int_{Jul}^{Feb}\sum_{n=0}^{N_{c}-1}p\left(n,t\right)dt \end{array}$$ (4)

Maximizing this objective is equivalent to maximizing the probability of flowering (i.e. having at least *N*_*c*_ active cells) in flowering season and non-flowering (i.e. having at most *N*_*c*_ active cells) during non-flowering season. The optimal reaction rates which maximized the objective led to time dependent optimal probability sequences which were capable of reproducing the idealized expression pattern of *FLC* that is switched off during flowering season. In Fig 4C, two different time points are chosen to show the typical probability distributions in flowering and non-flowering seasons. On 1st January, the most density (99.2%) of the probability located below the critical value of 30 cells, whereas on 15th April most density (92.2%) was distributed above the critical value. As shown in Fig 4D that the probability of having at least *N*_*c*_ active cells at different time of a yearly cycle preserved the idealized expression pattern of *FLC*, which was active during the non-flowering season and then gradually switched off in flowering season.

Under the condition that the autocorrelation length of temperature fluctuations in time decays exponentially, it was shown that the idealized expression patterns of *FLC* could be rebuilt from the stochastic model. By relaxing the autocorrelation condition, we would also like to investigate the effect of climate information on flowering time decision using machine learning, which is typically not requiring great details of the system thus can be more broadly applied to different climates.

### Long-term cold temperature and short-term day lengths together as a robust signal

To make flowering time decisions upon environmental cues such as temperatures and day lengths, plants are essentially information processing units for extracting critical environmental signals in order to survive by making the correct transit to the reproductive state. We employed artificial neural networks to approximate the information processing in plants by predicting the flowering season. The networks were trained to learn the idealized expression pattern of *FLC* from temperatures and day length from different climates, and the results relied on climate data in Cologne. The approach was broken into two tasks: to determine the effective memory length of determining season and to reconstruct the idealized expression pattern of *FLC* of *Arabidopsis* perennials.

The first step was to determine the number of days in the past that the plants need to remember in order to recognize the current season. It was cast into a classification problem as for given consecutive *L* days of temperature, the neural networks learned to classify which time window the temperature belonged to. The results showed that for Cologne, the prediction achieved MCC score of 0.964 for temperature memory of over 40 days. And increasing the memory length did not increase the score accordingly. The result was consistent with experimental result [5]. This reflected an expected tradeoff between sufficiently long memory to reduce the fluctuations of temperature signal (variance) and the loss of season specific averaged temperature if the memory stretches beyond the length of the season (bias). The detailed classification result can be found in S1 File.

**Fig 5:**
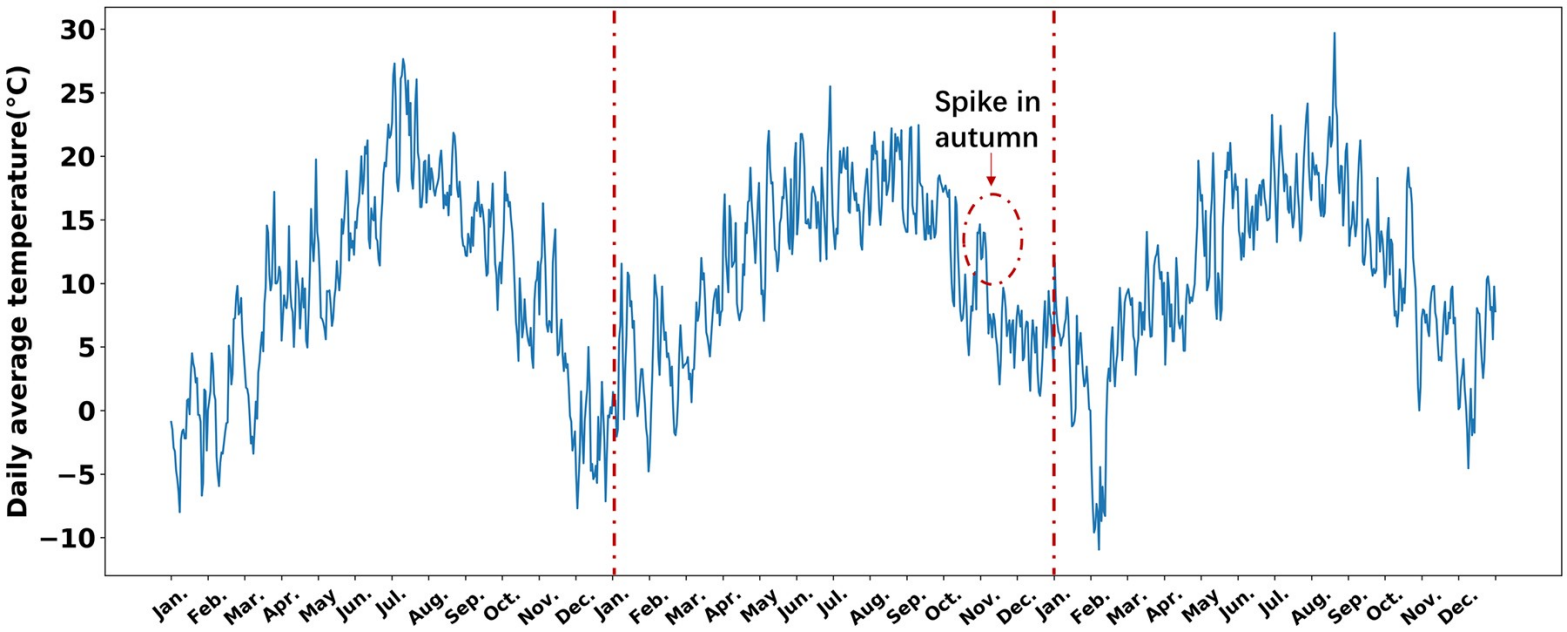
Three years of test temperatures. Three years of temperatures were used to test the regression models. In the second year, one can observe a long temperature spike starts from late October to early November. This is in correspondence with a predicted local minimum in Fig 6.

**Fig 6:**
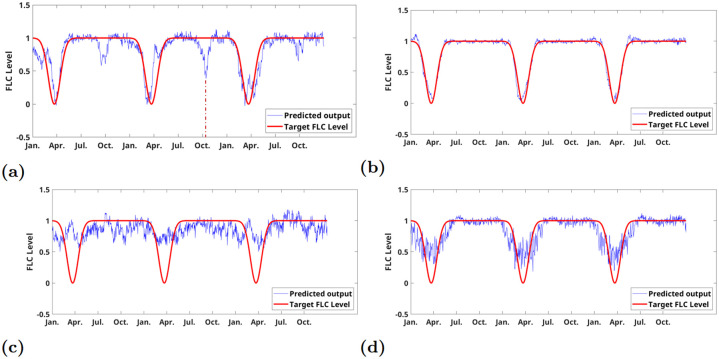
Predicted expression patterns by neural networks. (a): *Cologne*, fitting result of the idealized *FLC* expressions using 42 days of temperature as the input features gave a local minimum in September due to similarity between spring and autumn; (b): *Cologne*, fitting result from 42 days of temperature and 2 days of day lengths with eliminated local minima; (c): *Kahului*, fitting result from 42 days of temperatures; (d): *Kahului*, fitting result from 42 days of temperature and 2 days of day lengths.

Having determined the effective memory length, we were able to construct the input features of the neural networks for fitting the idealized expression pattern of *FLC* of *Arabidopsis* perennials. With only the temperature memory fed into the neural networks, the trained neural network was tested on three years of temperature as shown in Fig 5. Local minima arose in autumn as shown in Fig 6A, which resulted from the temperature similarity between spring and autumn. Especially for the second test year, a clear local minimum was observed in October, which has a correspondence to the high temperature spike in October as indicated in Fig 5. The local minima could potentially give a high chance of making wrong decision to flower in autumn. In order to remove the local minima and have a robust detection of spring, two days of day-length signal were added to the input features. It can be seen from Fig 6B that the local minima are eliminated, leading to more precise regression of the *FLC* expression pattern. The precise and robust reconstruction of the *FLC* signal was critical for precise flowering decisions in the right season. As a comparison, the climate data from Kahului, Hawaii was used as well to learn the *FLC* pattern. Fig 6C showed that the temperature memory only features led to a high-error fitting, which probably due to both the flat seasonal changes and the long autocorrelation in temperature fluctuations as shown in S4e Fig in S1 File. The integration of two days of day lengths increased the regression but still being noisy. The result may provide an explanation that vernalization in natural tropical climates was not established.

The neural networks based method was broadly applicable to different climates (the results for other climates are shown in the S1 File), unlike the stochastic model which required more system details and climate properties. Its fitting results from the climates data reflected that for temperate regions, long-term temperature and short-term day length together were deployed as a robust signal for determining the flowering transition, while for other regions, merely temperature and day length signals were not sufficient to have a mechanism such as the interplay between *FLC* and *FT*.

### Evolutionary simulation favors integration of temperature and day length

To investigate the possible effects of evolution on the role of temperature and day length in achieving the idealized expression pattern of FT, we conducted simulations using neural networks as plants’ agents. In each simulation, three groups of virtual plants were given access to either temperature, day length, or both. The Kullbach-Leibler Divergence (KLD) between the fitted and idealized expression patterns was used as the fitness measure. Fitnesses were calculated for individuals from each group and normalized to the population of each generation to have a fitness probability for each individual. Individuals then reproduced according to their fitness probability, that is, individuals with higher fitnesses had more offspring accordingly. Multiple offspring were possible for each individual, and offspring had access to the same input type as their parents (temperature, day length, or both). For each reproduced generation, a fixed number of mutations, represented by randomly selected neural network weights, were applied to each individual. Reproduction continued until 500 cycles were simulated or until all surviving individuals were the offspring of only one of the three groups. Each simulation was repeated 50 times, and the proportion of offspring per group throughout the entire simulation were tallied to estimate fixation probability.

**Fig 7:**
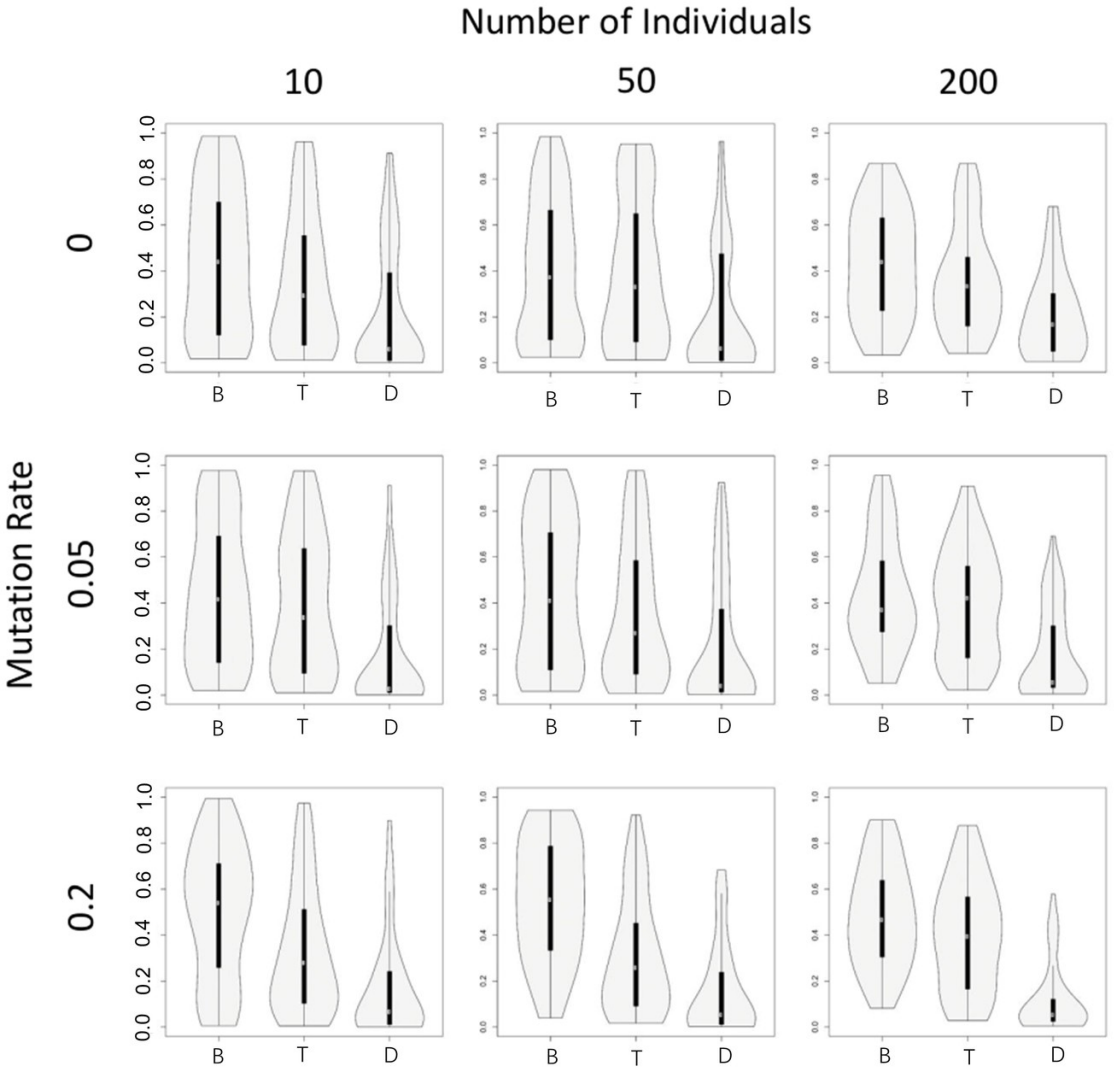
Evolution simulation. Distributions of group offspring proportions for mutation rates of 0, 0.05, and 0.2, for group sizes of 10, 50, and 200 individuals. “B” stands for group with access to both temperature and day length, “T” for temperature and “D” for day length.

To simulate the effects of strongly or weakly deleterious mutations, simulations were run with various numbers of mutations per generation. To investigate the effects of population size and genetic drift, the number of individuals per group was varied. The results of the simulations are shown in Fig 7, as violin plots of group offspring proportions under different mutation rates and population sizes.

The group with access to both temperature and day length generally had the most offspring, and the group with access to day length alone had the least. The temperature-only group performed somewhere between the two others. Consistent with the basic principles of genetic drift, larger population sizes resulted in less variance in the fixation probability. Possibly due to the small number of input variables in the neural network, the group with access to day length alone performed notably worse as the mutation rate increased.

## Conclusion

Our study verified that the temperature and day length in temperate regions like Cologne have the information premise for plants such as *Arabidopsis* to establish the epigenetic switch vernalization. For different climates, the vernalization requires different memory spans due to different temperature seasonal properties. The memory spans depended on the autocorrelations in temperature fluctuations, which decayed differently from climate to climate. For regions with flat temperature dynamics like San Fransisco and Hawaii, the autocorrelations decay slower than regions like Cologne, and have autocorrelation length above 100 days, which might be the reason to fail the establishment of vernalization. Our stochastic model, describing the dynamics of the number of cell with repressed *FLC*, was able to integrate the temperature dynamics as well as the temperature fluctuations. For regions with exponential decays in temperature fluctuation autocorrelations, the switch behavior of *FLC* in *Arabidopsis* perennials can be reconstructed. Further, without requirements on climate properties and system details, our machine learning approach showed that the idealized expression patterns of *FLC* can be robustly reconstructed by the combination of prolonged cold and short-term day lengths (e.g. the absolute day lengths from two consecutive days). The strategy of combining long-term cold and short-term day length is proven to be also favored by an evolution simulation where neural networks were regarded as the agents of plants for processing climate information.

Although in natural environments, temperate plants need to cope with other signals such as ambient temperature using additional genes like *FLM* [40], it might indicate the backbone of flowering mechanism is that the plants utilized long-term temperature to detect seasonal changes and used absolute day lengths to decide the eventual flowering days. The reason is that, for temperate climates, a cold winter is guaranteed and it is easier to track the absolute day length than the variations in day lengths which is 4min in maximum from day to day. It could also be a good strategy for cold regions like Norway since the autocorrelation decays similarly to Cologne, but due to lower temperature and shorter summer, the fast life cycles such as summer annuals of *Arabidopsis* was adapted [5]. In this case, ambient temperature and light intensity might play a more important role in plants’ vegetative or reproductive timing. In tropical regions where locate the most diverse and abundant plant species on earth, more factors have to be taken into consideration and merely temperature and day length are not sufficient for flowering decision making. For instance, flowering is mostly rain-season dependent, which might play a more critical role than the temperature and day length as they contain less seasonal information than that of temperate regions.

By investigating flowering decision making from an information point of view, our study suggested that, for temperate regions, cold winter memory and short term of day length can serve as a robust strategy for plants to determine flowering season.

## Supporting information

S1 File Details of data preprocessing, proofs of analytical solution to stochastic model, regression of the idealized *FLC* expressions based on different climates. (PDF)

S1 Code It includes the code *autocorrelation.py* for analyzing temperature data, *optimization.py* for optimizing the flowering objective, *evolution.py* for the evolutionary simulations and *neuralnets.m* for cleansing temperature data and training neural networks. (ZIP)

S1 Data It includes csv files for the temperature and day length data of Cologne, Norway, Auckland, Kahului and San Francisco. (ZIP)
