# Supplementary material for: Information integration and decision making in flowering time control
Source: PLoS One. 2020 Sep 23;15(9):e0239417. doi: 10.1371/journal.pone.0239417 (PMC7511014; doi:10.1371/journal.pone.0239417)
Supplement: S1 File — (PDF) [file pone.0239417.s001.pdf]

Information integration and decision making in flowering time control-Supplementary

Linlin Zhao[1,*], Sarah Richards[2], Franziska Turck[3], and Markus Kollmann[1,*]

[1]Institute of Mathematical Modeling for Biological Systems, Heinrich-Heine-University Dusseldorf, Dusseldorf, Germany
[2]Institute of Population Genetics, Heinrich-Heine-University Dusseldorf, Dusseldorf, Germany
[3]Max-Planck Institute for Plant Breeding Research, Cologne, Germany
[*]markus.kollmann@hhu.de, zhao@hhu.de

1 Data preprocessing

1.1 Source data cleaning

The data preparation is illustrated by using data from Cologne. The retrieved temperature data from NOAA includes recordings of daily maximal and minimal temperatures of 2 stations in Cologne. A snapshot of the customized CSV data file is shown in Fig. S1. Each station independently recorded daily temperatures. Thus, for Cologne, we have 98 years of daily temperature summaries (Table S1).

Noises of different sources are presented in the temperature data, for instance, missing dates or missing records in a day. We refer to the missing values as "holes" of the data. The dataset was cleansed as follows:

- For the simplicity of computation, each year was trimmed to 360 days and each month was modified to 30 days for the neural networks based models. For the stochastic model, yearly cycle of 365 days was used;
- The holes were filled by averaging neighbouring two days or just copying the neighbouring date if the hole was less than 10 days. For holes larger than 10 days, they were filled with the same dates from a nearby station;

	1	2	3	4
1	Station	Date	Daily max (x10)	Daily min (x10)
2	GHCND:GME00121042	19580101	88	64
3	GHCND:GME00121042	19580102	76	-36
4	GHCND:GME00121042	19580103	5	-76
5	GHCND:GME00121042	19580104	-2	-65
6	GHCND:GME00121042	19580105	84	-14
7	GHCND:GME00121042	19580106	120	-3
8	GHCND:GME00121042	19580107	115	16
9	GHCND:GME00121042	19580108	50	12
10	GHCND:GME00121042	19580109	83	42
11	GHCND:GME00121042	19580110	68	38

Figure S1: A snapshot of the customized CSV file retrieved from NOAA with 4 columns.

Data from the stations which have more than 20% of holes were discarded.

The day length data of Cologne were downloaded from `ptaff.ca`. The orbit of the earth around the sun is stable such that the day length variation remains the same from year to year. Thus we just replicated the yearly day length for Cologne to 98 years. Meanwhile, to count influence of weather conditions on day lengths, Gaussian noises with adjustable strength were added to different years.

The same cleaning procedure was applied to other climates. The datasets of all retrieved climates are summarized in Table S1.

Table S1: Number of Years temperature for different regions

Regions	Cologne	Oslo	Kahului	Auckland	San Francisco
Number of Years	98	57	237	421	154

2 Time series of temperature data

2.1 Decomposing and analyzing temperature data

The temperature data from Cologne were analyzed. The consecutive daily recordings of temperatures are time series data where neighbouring days of temperatures are correlated. For instance, a storm brings rains as well as temperature drops. The dropped temperatures are usually correlated for a few days. The changes in temperatures due to weather conditions are termed temperature fluctuations and the seasonal temperature changes are termed deterministic temperature dynamics. The temperature fluctuation on day t can be mathematically described as

$$\delta T(t) = T(t) - (\bar{T} + \langle T(t) \rangle)$$

where $T(t)$ denotes the real temperatures, $\langle T(t) \rangle$ is the deterministic temperature dynamics with period of 365 days and $\bar{T}$ is the arithmetic average of temperature. The daily average temperature is obtained by averaging the maximal and minimal temperatures. The 98 years of temperature recordings from Cologne as

$$\text{Temperature fluctuations of each year} = \text{Each of 98 years} - (\langle T(t) \rangle + \bar{T}).$$

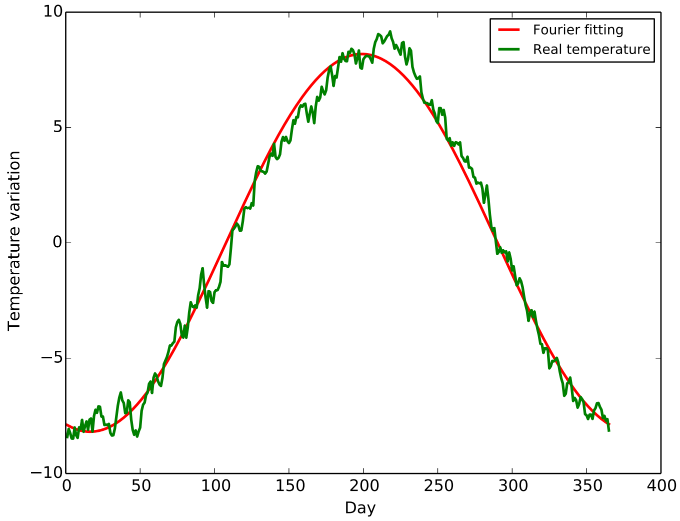

Figure S2: The real temperature is obtained from averaging 98 years of temperatures in Cologne. The fitted temperature curve is based on a second order Fourier series.

Fourier series based regression for $\langle T(t)\rangle$

To mimic the long term adaptation of plants to local climates, the average of 98 years of temperature over one-year period gives an estimate of temperature dynamics and averaged temperature fluctuations. The temperature dynamics was fitted by a second-order Fourier series as

$$\langle T(t)\rangle = a_0 + a_1\cos(wt) + c_1\sin(wt) + a_2\cos(2wt) + c_2\sin(2wt) \tag{1}$$

with determined parameters

$$(a_0, a_1, c_1, a_2, c_2, w)_T = (0, -7.775, -2.669, -0.1552, 0.4052, 0.0172). \tag{2}$$

The parameterization of the fitting was performed using Riemann sum [1]. The fitted result is shown in Fig. S2.

The exponential decay in temperature time series

The autocorrelation in temperature fluctuations is defined as

$$R(t) = \frac{1}{n-k}\sum_{t=1}^{n-k}\delta T_t \delta T_{t+k}, \tag{3}$$

where n is the total number of days, and k is the presumed maximal correlated number of days, which was set as 80 in our calculation. It is shown in Fig. S3a that the autocorrelations in temperature fluctuations follow an exponential decay which can be fitted

as

$$autocorrelation(t) = \sigma^2 e^{-t/\tau}, \tag{4}$$

where $\sigma^2 = 12.67$ and $\tau^{-1} = 0.22$ with a fitting R^2 score of 0.934. The least square method implemented in SciPy [2] was used to fit the exponential decay. In addition, we performed a bootstrap analysis to estimate the confidence interval of the fitted coefficients. Since the fluctuations are correlated, in order to preserve the correlation, the sampling used a block length of 50 days. From the bootstrap with 10000 samples, the confidence intervals for σ^2 and τ were estimated as $(11.90, 13.42)$ and $(0.199, 0.253)$ respectively. The fitted coefficients indeed located in the confidence interval. The bootstrapped fitting results are shown in Fig S3b.

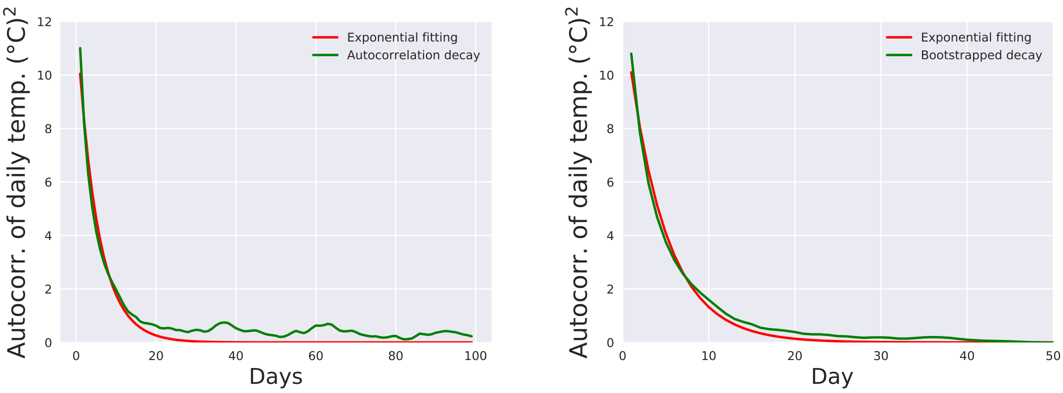

(a) (b)

Figure S3: The real decay of autocorrelations exhibits random fluctuations after about 15 days. The exponential decay fitting smoothed these fluctuations to zero.

2.2 Autocorrelation analysis for other climates

Similar to the calculation of autocorrelation in temperature fluctuations for Cologne, the procedure was applied to other selected climates as shown in Fig S4. It can be observed that the autocorrelations for Cologne, Oslo and Auckland showed exponential decays but with different lower bounds, which may be due to different noise levels in temperatures. And for regions with less seasonal changes like Hawaii and San Francisco, exponential decays were not observed.

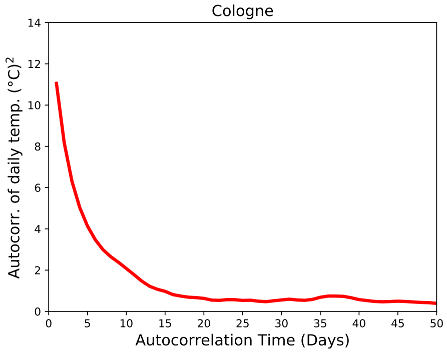

(a)

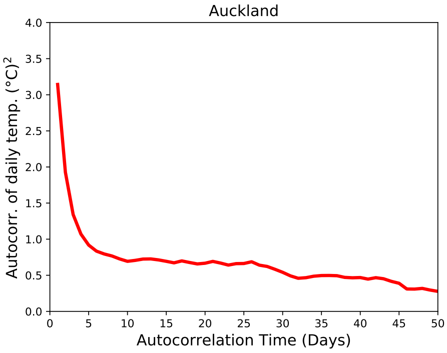

(b)

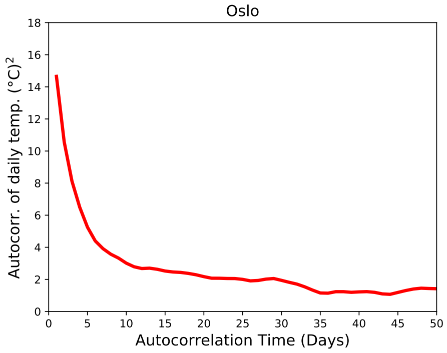

(c)

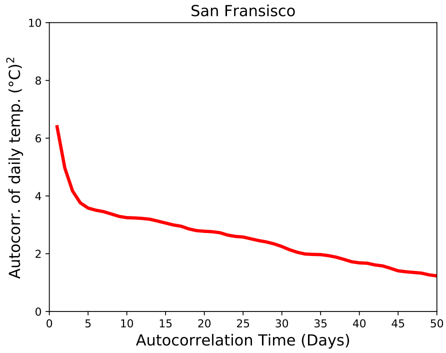

(d)

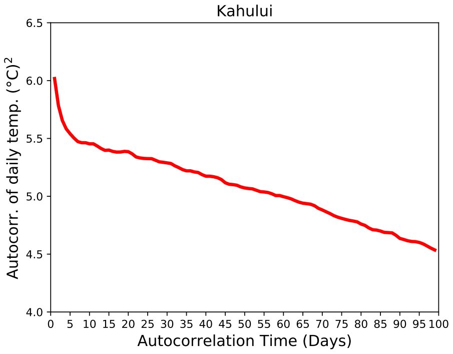

(e)

Figure S4: The autocorrelation time decays for different climates.

3 Modeling the process of producing active cells

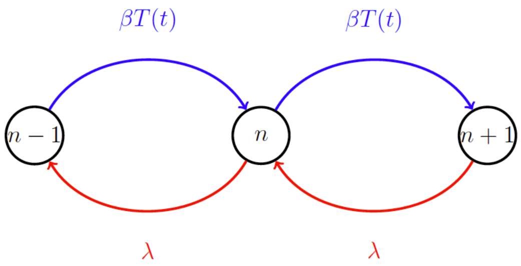

Figure S5: Temperature-driven birth-death process of cells with active histone modifications. n stands for the number of actively modified cells, the production rate $\beta T(t)$ depends on the temperature $T(t)$ and λ is the degradation rate.

3.1 The master equation and its analytical solution

Following the previous work of modeling simple birth-death process for one species [3, 4, 5], the process of having n cells with active modifications is simplified to a reaction of the form $\phi \underset{\lambda}{\overset{\beta T(t)}{\rightleftharpoons}} \mathrm{M}$ which is further illustrated in Fig. S5. The production rate $\beta T(t)$ depends on the temperature $T(t)$ and λ is the degradation rate of active cells. The total number of cells N was reduced 60 for computation feasibility. The master equation for describing the reaction can be written as

$$\partial_t p(n,t) = \beta T(t)\big(p(n-1,t) - p(n,t)\big) - \lambda n p(n,t) + \lambda(n+1)p(n+1,t). \tag{5}$$

To solve the partial differential equation, we first need to introduce the time-dependent probability generating function $G(s,t) = \sum_{n=0}^{\infty} s^n p(n,t)$. Then multiplying s^n to the equation (5) and summing over n can transform the equation to

$$\partial_t G(s,t) = \beta T(t)(s-1)G(s,t) - \lambda(s-1)\partial_s G(s,t). \tag{6}$$

First neglecting the time dependence of the temperature, the solution of the equation (6) is

$$G(s,t) = (1 + (s-1)e^{-\lambda t})^N e^{\beta T(s-1)\int_0^t e^{-\lambda t'} dt'} \tag{7}$$

(the derivation of the solution can be found in chapter 7 of [4]). Now considering the time dependence of T, we can assume the solution becomes

$$G(s,t) = \big(1 + (s-1)e^{-\lambda t}\big)^N e^{\beta(s-1)F(t)}. \tag{8}$$

To find $F(t)$, substituting (8) into (6) yields

$$F'(t) + \lambda F(t) = T(t). \tag{9}$$

By solving the differential equation (9), we have

$$F(t) = ce^{-\lambda t} + e^{-\lambda t} \int_0^t T(t')e^{\lambda t'} dt'. \tag{10}$$

Under the assumptions of $c = 0$ and $T(t) = d + \langle T(t)\rangle + \delta T(t)$ where d is the constant for converting temperature in Celsius to Kelvin, $\langle T(t)\rangle$ is the temperature average over years and $\delta T(t)$ is the temperature fluctuation, the equation (8) can be written as

$$G(s,t) = \left(1 + (s-1)e^{-\lambda t}\right)^N e^{\beta(s-1)e^{-\lambda t} \int_0^t \left(d + \langle T(t')\rangle + \delta T(t')\right) e^{\lambda t'} dt'}. \tag{11}$$

Considering the stationary state of $G(s,t)$, which follows a yearly cycle and is still time dependent, we have

$$G^*(s,t) = e^{\frac{d\beta}{\lambda}(s-1)} \left\langle e^{\beta(s-1)e^{-\lambda t} \int_{-\infty}^t \delta T(t')e^{\lambda t'} dt'} \right\rangle e^{\beta(s-1)e^{-\lambda t} \int_{-\infty}^t \langle T(t')\rangle e^{\lambda t'} dt'} \tag{12}$$

Noise-free temperature

Now, starting with the simple scenario, we assume temperature dynamics are noise-free, which means $\delta T(t) = 0$. Consequently, the steady state solution becomes

$$G^*(s,t) = e^{\frac{d\beta}{\lambda}(s-1)} e^{\beta(s-1)e^{-\lambda t} \int_{-\infty}^t \langle T(t')\rangle e^{\lambda t'} dt'} \tag{13}$$

Zooming into the equation (13), we need to deal with the part related to temperature dynamics: $e^{-\lambda t} \int_{-\infty}^t \langle T(t')\rangle e^{\lambda t'} dt'$. For the simplicity of notation, we define

$$D(\lambda, t) := e^{-\lambda t} \int_{-\infty}^t \langle T(t')\rangle e^{\lambda t'} dt'. \tag{14}$$

For the data collected from Cologne, the temperature dynamics $\langle T(t)\rangle$ can be numerically approximated by a second-order Fourier series (Fig. S2). Using the parameters fitted in the approximation (1), we have

$$\begin{aligned} D(\lambda,t) = \frac{a_0}{\lambda} + \frac{a_1}{w^2+\lambda^2}\left(\lambda\cos(wt) + w\sin(wt)\right) + \frac{c_1}{w^2+\lambda^2}\left(\lambda\sin(wt) - w\cos(wt)\right) + \\ \frac{a_2}{(2w)^2+\lambda^2}\left(\lambda\cos(2wt) + 2w\sin(2wt)\right) + \frac{c_2}{(2w)^2+\lambda^2}\left(\lambda\sin(2wt) - 2w\cos(2wt)\right), \end{aligned}$$

where $t \in [0, 2L]$ with $2L$ the period of temperature cycle, and coefficients were determined in section 2. By further defining $b := \frac{d\beta}{\lambda} + \beta D(\lambda, t)$, the stationary generating function becomes

$$G^*(s,t) = e^{b(s-1)}, \tag{15}$$

which is a generating function for a Poisson distribution. Therefore, in the case of noise-free temperatures, the stationary solution to the master equation (5) is

$$p(n,t) = \frac{b^n}{n!}e^{-b}. \tag{16}$$

Noisy real temperature

Now considering the real noisy temperature, we need to deal with the part caused by temperature fluctuations: $\left\langle e^{\beta(s-1)e^{-\lambda t}\int_{-\infty}^{t}\delta T(t')e^{\lambda t'}dt'}\right\rangle$. Since the fluctuations has been shown to have an exponential decay in autocorrelations for temperatures in Cologne, by Doob's theorem [6], they follow a Gaussian Markovian Process. Therefore, we have

$$\left\langle e^{\beta(s-1)e^{-\lambda t}\int_{-\infty}^{t}\delta T(t')e^{\lambda t'}dt'}\right\rangle = e^{\frac{1}{2}\beta^2(s-1)^2\int_{-\infty}^{t}\int_{-\infty}^{t}\left\langle \delta T(t_1)e^{\lambda(t_1-t)}\delta T(t_2)e^{\lambda(t_2-t)}\right\rangle dt_1 dt_2} \quad (17)$$

$$= e^{\frac{1}{2}\beta^2(s-1)^2\int_{-\infty}^{t}\int_{-\infty}^{t}\left\langle \delta T(t_1)\delta T(t_2)\right\rangle e^{\lambda(t_1+t_2-2t)}dt_1 dt_2} \quad (18)$$

$$= e^{\frac{1}{2}\beta^2(s-1)^2\int_{-\infty}^{t}\int_{-\infty}^{t}\sigma^2 e^{-\frac{|t_2-t_1|}{\tau}}e^{\lambda(t_1+t_2-2t)}dt_1 dt_2} \quad (19)$$

$$= e^{\frac{\beta^2\sigma}{2\lambda(\lambda+A)}(s-1)^2}. \quad (20)$$

With $a := \frac{\beta^2\sigma^2}{2\lambda(\lambda+\tau^{-1})}$ and $b = \frac{d\beta}{\lambda} + \beta D(\lambda, t)$, the stationary $G(s,t)$ (12) can be written in a closed form as

$$G^*(s,t) = e^{a(s-1)^2+b(s-1)}. \quad (21)$$

Due to the quadratic term in the exponential of (21), finding the associated probability density function requires the introduction of an auxiliary generating function which can be expanded in terms of Hermite Polynomial

$$G_a(x,t) = e^{-t^2+2xt} = \sum_{n=0}^{\infty} H_n(x)\frac{t^n}{n!}. \quad (22)$$

By changing variables $a = -\alpha^2$, $t = \alpha s$ and $x = \frac{b+2\alpha^2}{2\alpha}$, we have the following deduction

$$G^*(s,t) = e^{b(s-1)+a(s-1)^2} = e^{a-b}e^{-\alpha^2 s^2+(b+2\alpha^2)s} = e^{a-b}e^{-t^2+2xt} = e^{a-b}\sum_{n=0}^{\infty} H_n(x)\frac{t^n}{n!}. \quad (23)$$

In short, the generating function (21) becomes

$$G^*(s,t) = e^{a-b}\sum_{n=0}^{\infty} H_n(x)\frac{t^n}{n!}. \quad (24)$$

Substituting the variables a and b to (24), we have

$$G^*(s,t) = e^{a-b}\sum_{n=0}^{\infty} H_n\left(\frac{b+2\alpha^2}{2\alpha}\right)\frac{(\alpha s)^n}{n!} = \sum_{n=0}^{\infty} s^n p(n,t). \quad (25)$$

Finally the probability density function is derived as

$$p(n,t) = e^{a-b}\frac{\alpha^n}{n!}H_n\left(\frac{b+2\alpha^2}{2\alpha}\right). \quad (26)$$

3.2 Optimization

The model parameters can be uniquely determined by optimizing a proper decision function in such a way that the signal-to-noise ratio determines the degradation rate and the decision boundary determines the production rate. When the degradation rate λ is very small, in order to have a reasonable distribution over different number of active cells, the reaction rate has to be very small too. In this slow reaction scenario, the effect of temperature dynamics is diluted, which make the reaction hard to capture the useful information in temperature. On the contrary, if λ is very large and β should be very large to maintain a certain number of active cells. In the fast production and degradation scenario, every single fluctuation in temperatures would drive the reaction in an undesirable manner. Both extreme cases are not practical for plants to rely on. Therefore, λ has to be tuned by the signal-to-noise ratio in temperature to a reasonable level. Meanwhile, the optimal value of β, λ can be determined by setting the decision boundary to a suitable number of active cells.

With the obtained parameters $d = 283.3K$, $\tau^{-1} = 0.22$, $\sigma^2 = 12.67$ for the exponential decay in temperature fluctuations and the parameters for the Fourier fitting of temperature dynamics in (2), the probability distribution $p(n, t)$ (26) has only the undetermined reaction rates β, and λ. By optimizing the following objective

$$F(\beta, \lambda) = \int_{Mar}^{Jun} \sum_{n=N_c}^{N_{max}} p(n, t)dt + \int_{Jul}^{Feb} \sum_{n=0}^{N_c-1} p(n, t)dt, \tag{27}$$

the optimal β and λ were obtained as

$$\beta = 6.45, \lambda = 2.94.$$

4 Artificial Neural Networks

A feedforward neural network with two layers is illustrated in Fig. S6, which can be mathematically described as

$$F_k(\mathbf{x}, W) = f\left(\sum_{j=1}^{M} w_{kj}^{(2)} h\left(\sum_{i=1}^{d} w_{ji}^{(1)} x_i + w_{j0}^{(1)}\right) + w_{k0}^{(2)}\right), \tag{28}$$

where $F_k(\mathbf{x}, W)$ denotes the kth output, d, M denote the dimension of input $\mathbf{x}$ (number of nodes in the input layer) and number of nodes in the hidden layer (second layer) respectively. $W = \left\{W^{(1)}, W_k^{(2)}\right\}$ where $W^{(1)}$ is the weight matrix with $w_{ji}^{(1)}$ connecting ith input to jth node in the hidden layer, $W_k^{(2)}$ is the weight matrix with $w_{kj}^{(2)}$ connecting jth hidden node to kth output node. In the context of regression, the output is usually one-dimensional, the index k can be dropped.

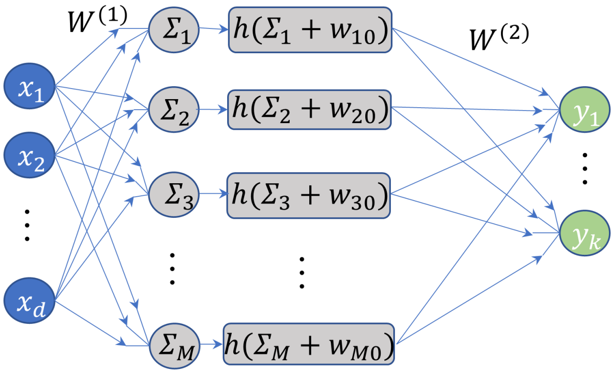

Figure S6: A fully connected feedforward neural network.

For a given *i.i.d.* dataset $\mathcal{D}$: $\mathbf{x}_i \in R^d$, $y_i \in R$, $i \in 1, 2, \cdots, N$, assuming that the targets y have a Gaussian distribution centered on $F(\mathbf{x}, W)$ gives rise to

$$p(y \mid \mathbf{x}, W) = \mathcal{N}(y \mid F(\mathbf{x}, W), \beta^{-1}), \tag{29}$$

with β^{-1} the variance of Gaussian noise. With defining the mean squared error between the targets and their model estimates as

$$\mathcal{E}(W) = \frac{1}{N} \sum_{i=1}^{N} \|F(\mathbf{x}_i, W) - y_i\|^2, \tag{30}$$

the likelihood function can be written as

$$\mathcal{L}(W) = p(y \mid \mathbf{x}, W) = \prod_{i=1}^{N} p(y_i \mid \mathbf{x}_i, W, \beta) = \frac{\exp(-\beta \mathcal{E}(W))}{Z(\beta)} \tag{31}$$

where $Z(\beta) = (2\pi/\beta)^N$. Taking negative logarithm of (31) gives

$$-\ln \mathcal{L}(W) = \mathcal{E}(W) + c, \tag{32}$$

where c is a constant related to β which can be neglected for the purpose of minimizing negative logarithm likelihood. The equation (32) shows that minimizing error function is equivalent to maximizing the likelihood function.

Consequently adding L_2 norm regularization leads to the final loss function as

$$Loss(W \mid \mathbf{x}, y) = \mathcal{E}(W) + \Omega(W) \tag{33}$$

$$= \frac{1}{N} \sum_{i} \|F(\mathbf{x}_i, W) - y_i\|^2 + \rho \parallel \mathbf{w} \parallel^2 . \tag{34}$$

5 Classification for estimating memory length

The input features are $(x_{l1}, \cdots, x_{lk}, x_{h1}, \cdots, x_{hk})$, where k is window length, x_{li} and x_{hi}, $i = 1, \cdots, k$, stand for daily lowest and highest temperatures respectively. For

example, when the window length is set to be $k = 30$ (i.e. a month), without loss of generality, April is set to be class "1", the rest months are set to be class "0". Then for the temperatures of each year, the corresponding targets are $(0, 0, 0, 1, 0, 0, 0, 0, 0, 0, 0, 0)$. The same configuration can be applied to other window lengths. We have tested lengths $k \in \{28, 42, 60\}$ by using a repeated cross validation routine, that is, for each model training, 80 years was randomly selected for training and the rest 18 years was used for testing. The averaged testing results were shown in Fig.S7. For length $k = 30$, the false positive rate is 26.3%, although the overall accuracy is 93.3%. When the length is increased to $k = 42$, the false positive rate dropped to zero, indicating that this length provides sufficient information for determining the season, which can be used as the effective memory length of plants for recognizing seasons.

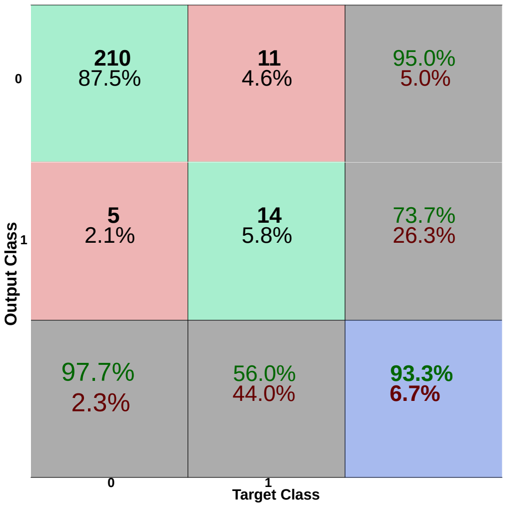

(a) 28 days

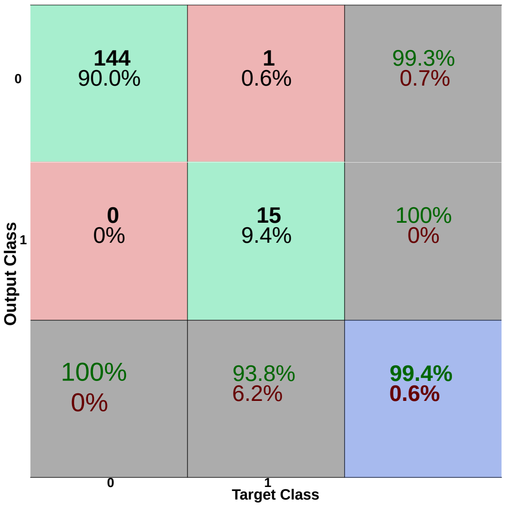

(b) 42 days

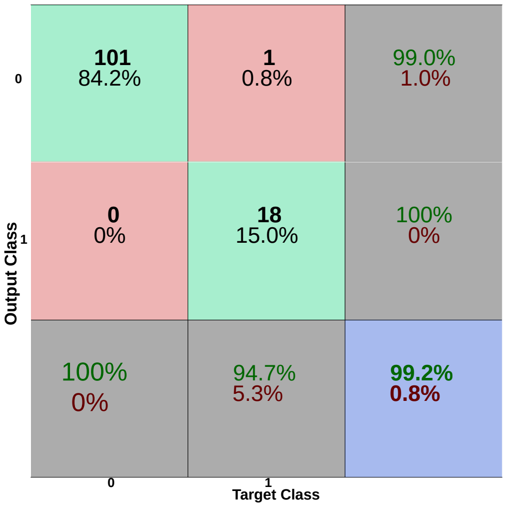

(c) 60 days

Figure S7: The confusion matrices for the classification of different temperature windows. The temperature data from Cologne was used. The typical window sizes of 4 weeks and 6 weeks were used to test the effective memory length. It was also shown that window size of 60 days did not improve the classification performance.

Regression of idealized expression patterns for various climates

In this section, different climates information was used to fit the idealized expression pattern of *FLC*. Based on the classification results, the input features and targets for regression were constructed as following. Each day of a year has a corresponding ex-

pression value from the idealized expression curve as the target. The features for that day comprised of the temperatures (daily maxima and minima) and/or day lengths of its precedent days. The number days for temperatures was taken as the effective memory length from the classification. For temperate region, long term memory of temperatures played a key role in the regression and the addition of day lengths significantly improved the regression.

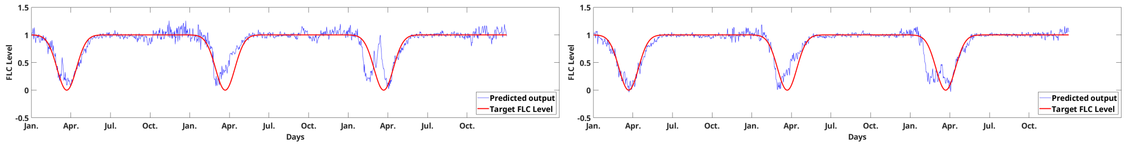

(a) Oslo, temperature only (b) Oslo, temperature and day lengths

Figure S8: The regression results of using different input features combinations from Oslo.

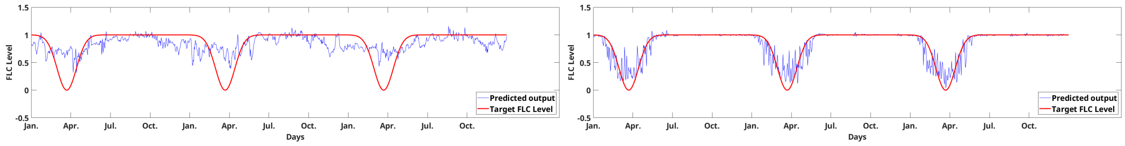

(a) San Francisco, 42 days of temperatures as input feature.

(b) San Francisco, 42 days of temperatures and 2 days of day lengths.

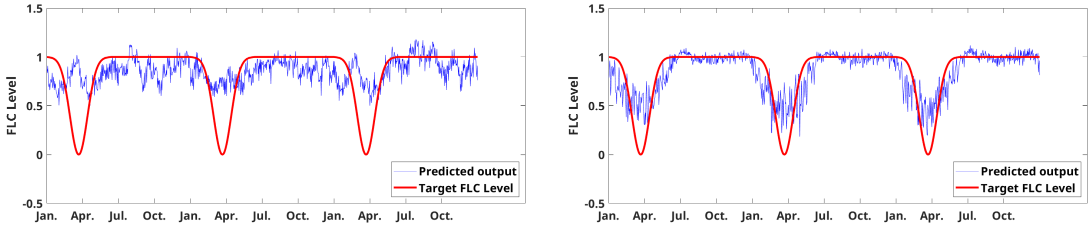

(c) Kahului, 42 days of temperatures as input features.

(d) Kahului, 42 days of temperatures and 2 days of day lengths.

Figure S9: The regression results by using data from regions with less seasonal changes.

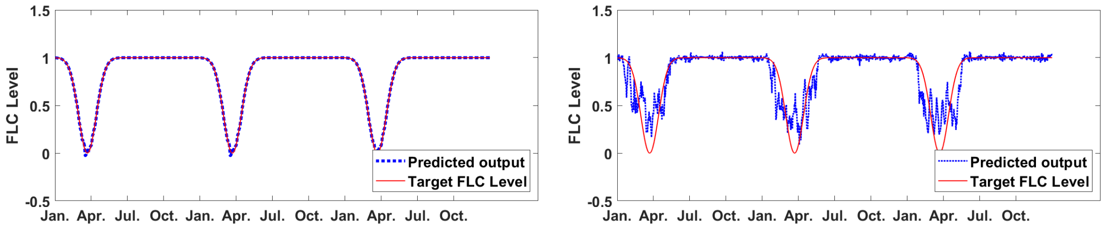

(a) Cologne, 7 days of day length (b) Kahului, 7 days of day length

Figure S10: The fitting results of using only 7 days of day length for both Cologne and Hawaii. The fitting for Cologne is almost perfect.

Regions as San Francisco and Kahului have very similar temperature every year, which means very few information can be extracted from their temperature. This also led to the poor generalization in predicting the idealized expression patterns (Fig S9). Moreover the predicting results showed no significant differences for different temperature memory lengths, e.g. 7 days, 20 days and 40 days. The results agree with that plants in tropical region, which do not rely on long term memory of temperature for flowering decision making. In order to better fit *FLC* expression patters of regions such as San Francisco and Kahului, temperature memory was reduced to at most one and longer term of day lengths were added as input features. For both San Francisco and Kahului, it was seen that the trained models with only temperatures as features generalized poorly. But the fitting accuracy can be improved by adding day lengths.

For the sake of comparison, the fitting results of using only day length were shown in S10 for Cologne and Hawaii. For both regions, 7 days of day length which were added with the same level of Gaussian noises were used. It can seen that the fitting for Cologne was better than that of Kahului, Hawaii. This may be due to the flatter day length dynamics in Hawaii than that in Cologne.

6 Evolution Simulation

We used neural networks as plant agents to simulate their adaptations to three settings of information, only temperature, only day length or both. Neural networks consist of multiple layers of neurons which can be regarded as decision makers. Individual neurons weigh the evidences which they receive from upstream neurons and output decisions using activation functions (e. g. sigmoid, linear or other functions) [7]. On the other hand, although gene regulatory networks in plants are very complex molecular systems, individuals genes usually take actions based on the regulations that they receive. The relation between gene production rate and transcription factor concentrations can be modeled by Hill function [8]. With proper Hill coefficients, the Hill function may behave similarly to the sigmoid function. Therefore we hypothesized that neural networks can serve well as plant agents in terms of investigating information from temperature and day length data. The simulation algorithm was summarized in Fig. S11. The Kullbach-Leibler divergence between the idealized expression pattern and the neural network-predicted expression level was used as the fitness for individuals. The individual reproduction rate was depended on their fitnesses. The mutation of offsprings was realized by mutating the weights of the neural network agents. More specifically, it followed three steps:

- Randomly select the specified number of weights from individual networks.
- Calculate the mean of all weights of the network.
- The mutation rate is applied to the calculated mean (mutationRate * Mean). The product is the used as the standard deviation from applying Gaussian distributed mutations to the selected weights.

The key parameters in the simulation are explained as following:

- indPerGroup: number of individuals with access to temperature, day length, or both);
- evCyc: number of evolution cycles, fixed as 500;
- Ntrain: number of training years for individual networks, 15 years was used in the final results;
- Ntest: number of testing years for evaluating Kullbach-Leibler divergence as fitness measure, 3 years was used in the final results;
- Nmut: number of weights from individual networks for mutation, the weights were randomly selected from all weights of individual networks, 5 weights was used in the final results;
- benRate: the learning rate of individual networks to adapt to new input data after mutation, 0.05 was finally used;
- mutRate: the standard deviation of Gaussian distribution which is used for mutating weights;
- rep: repetition number of simulation, 50 was used.

The simulation results with different mutation rates and indPerGroup were shown in Fig. S12.

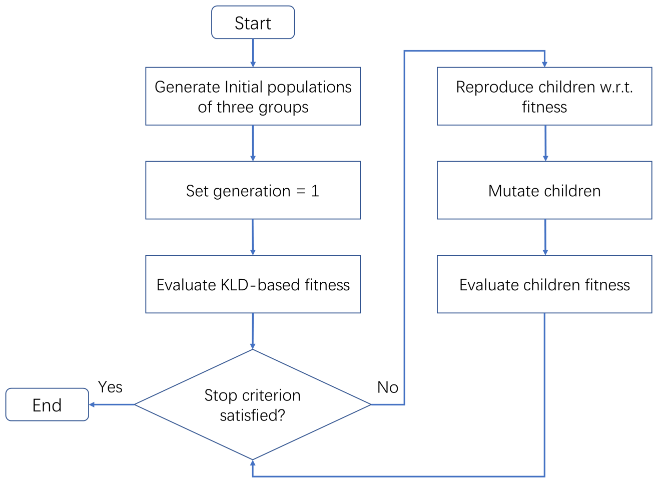

Figure S11: Evolutionary simulations for each group of the population.

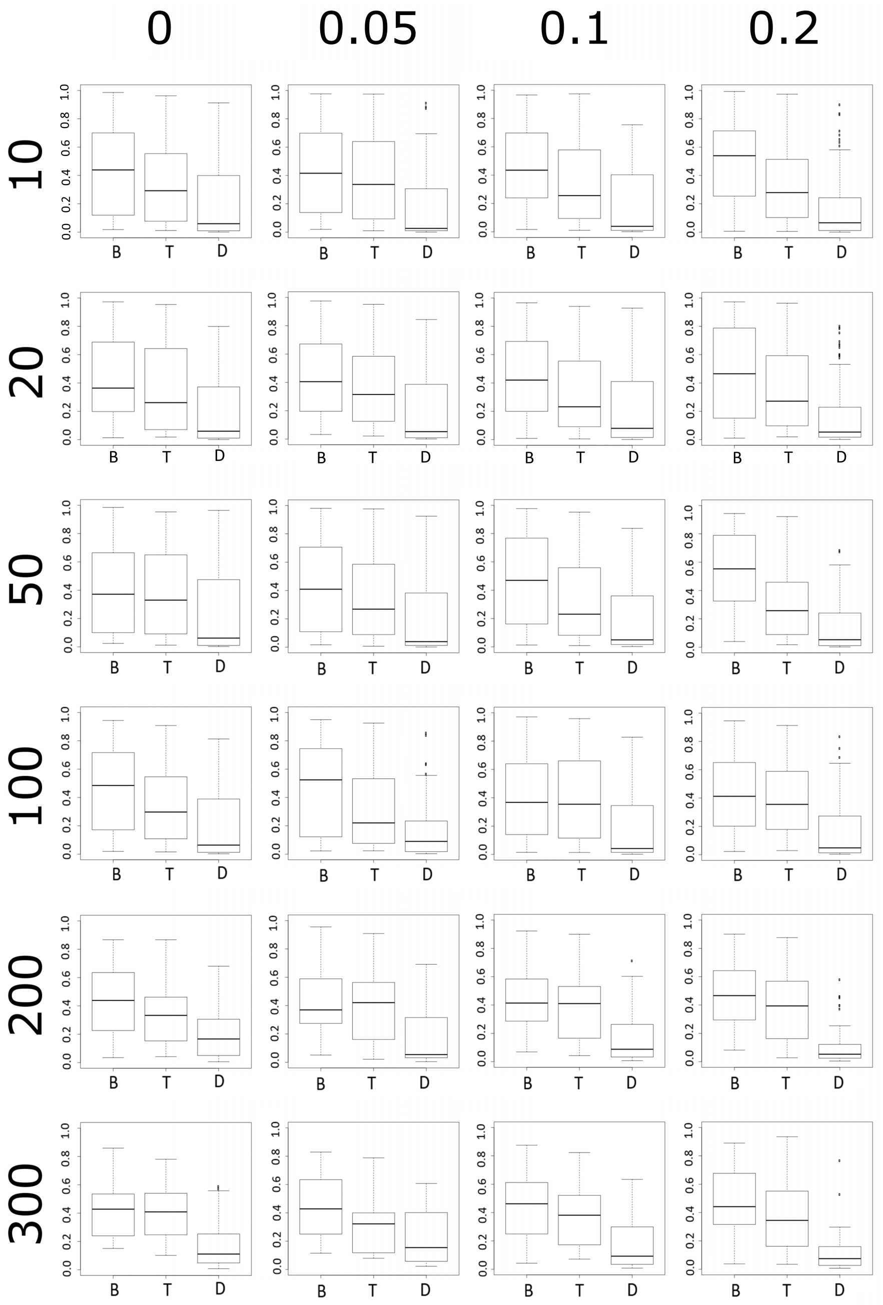

Figure S12: The proportion of survived individuals for different groups. The vertical axis of the plot represents the group sizes, and the horizontal axis represents the mutation rates. Each subplot has three boxplots which corresponding to the distribution of the proportions of survived individuals with accesses to temperatures, day lengths or both.

References

[1] Mermoz. How to calculate a Fourier series in Numpy?;. Stack Overflow. Available from: https://stackoverflow.com/questions/4258106/.

[2] Jones E, Oliphant T, Peterson P, et al.. SciPy: Open source scientific tools for Python; 2001–. Available from: http://www.scipy.org/.

[3] Walczak, A. M., Mugler, A. & Wiggins, C. H. Analytic methods for modeling stochastic regulatory networks. In *Computational Modeling of Signaling Networks*, 273–322 (Springer, 2012).

[4] Gardiner, C. W. Handbook of stochastic methods: for physics, chemistry and the natural sciences. (2004).

[5] Rausenberger, J. & Kollmann, M. Quantifying origins of cell-to-cell variations in gene expression. *Biophysical journal* **95**, 4523–4528 (2008).

[6] Doob, J. L. The brownian movement and stochastic equations. *Annals of Mathematics* 351–369 (1942).

[7] Nielsen M. Neural Network and Deep Learning. Available from: http://neuralnetworksanddeeplearning.com/index.html.

[8] bio-physics wiki. Hill function;. Available from: http://www.bio-physics.at/wiki/index.php?title=Hill_Function.
